# The Proteome and Secretome of Cortical Brain Cells Infected With Herpes Simplex Virus

**DOI:** 10.3389/fneur.2020.00844

**Published:** 2020-08-27

**Authors:** Niko Hensel, Verena Raker, Benjamin Förthmann, Anna Buch, Beate Sodeik, Andreas Pich, Peter Claus

**Affiliations:** ^1^Institute of Neuroanatomy and Cell Biology, Hannover Medical School, Hanover, Germany; ^2^Niedersachsen-Research Network on Neuroinfectiology (N-RENNT), Hanover, Germany; ^3^Center for Systems Neuroscience (ZSN), Hanover, Germany; ^4^Institute of Virology, Hannover Medical School, Hanover, Germany; ^5^DZIF—German Centre for Infection Research, Partner Site Hannover-Braunschweig, Hanover, Germany; ^6^Institute for Toxicology, Hannover Medical School, Hanover, Germany; ^7^Core Facility Proteomics, Hannover Medical School, Hanover, Germany

**Keywords:** herpes simplex virus-1, encephalitis, NEK7, inflammasome, Rab GTPases, ArfGap1, glutathione, semaphorin

## Abstract

Infections of the brain with herpes simplex virus type 1 (HSV-1) cause life-threatening Herpes simplex encephalitis (HSE) characterized by viral replication in neurons and neuro-inflammation including an infiltration of peripheral immune cells. HSV-1 reprograms host cells to foster its own replication and for immune evasion, but eventually the immune responses clear the infection in most patients. However, many survivors suffer from long-term neuronal damage and cannot regenerate all brain functions. HSV-1 influences the physiology of neurons, astrocytes, oligodendrocytes and microglia, and significantly changes their protein expression and secretion pattern. To characterize temporal changes upon HSV-1 infection in detail, we inoculated mixed primary cultures of the murine brain cortex, and performed quantitative mass spectrometry analyses of the cell-associated proteome and the secretome. We identified 28 differentially regulated host proteins influencing inflammasome formation and intracellular vesicle trafficking during endocytosis and secretion. The NIMA-related kinase 7 (NEK7), a critical component of the inflammasome, and ArfGap1, a regulator of endocytosis, were significantly up-regulated upon HSV-1 infection. In the secretome, we identified 71 proteins including guidance cues regulating axonal regeneration, such as semaphorin6D, which were enriched in the conditioned media of HSV-1 infected cells. Modulation of inflammasome activity and intracellular membrane traffic are critical for HSV-1 cell entry, virus assembly, and intracellular spread. Our proteome analysis provides first clues on host factors that might dampen the inflammasome response and modulate intracellular vesicle transport to promote HSV infection of the brain. Furthermore, our secretome analysis revealed a set of proteins involved in neuroregeneration that might foster neuronal repair processes to restore brain functions after clearance of an HSV-1 infection.

## Introduction

Herpes simplex virus (HSV) infections of the brain may lead to severe herpes simplex encephalitis (HSE); one of the most common causes for infectious encephalitis (1–3). HSV-1 is mostly responsible for HSE with only a minor fraction being associated with HSV-2 (4, 5). HSE lethality ranges from 5 to 20%, and the survivors often suffer from long-term disabilities including memory deficits, personality changes and psychiatric disorders (1). This is in line with the infected brain regions since HSV-1 commonly localizes to cortical regions such as the frontal and temporal lobes (6).

Sixty to ninety percent of the adult population worldwide harbor antibodies against HSV-1 classifying it as one of the most prevalent human viruses (7, 8). Fortunately, HSV infection of the brain is a rare event with an annual HSE-incidence of 1 to 2 in 500,000 (3). HSV-1 initially infects epithelial cells and fibroblasts, enters free nerve endings in the skin or mucosal membranes and is transported retrogradely to the trigeminal ganglion where it establishes life-long latency (9). Stress may trigger reactivation from latency with the production of progeny virions which are transported back to the periphery where they cause recurrent herpes labialis (10, 11). The initial mechanism of viral entry to the brain upon primary or recurrent infections are not well-understood despite many years of animal and human research (3).

HSV-1 coevolved with humans and developed specific strategies to replicate within the host, to evade the host immune responses, and to spread globally within the human population (12, 13). Given the rareness of an HSE and its high mortality, HSE patients contribute little to HSV-1 spread of HSV-1 among humans, and brain infections exert very little evolutionary pressure on HSV-1 strains. Once HSV-1 enters the brain parenchyma, host immune responses which are tightly controlled during latent infection, become activated to limit the viral spread and tissue damage. Accordingly, HSE patients under immunosuppression have the same susceptibility but an enhanced mortality compared to non-immunosuppressed patients (14, 15). While host immune responses in the brain are crucial for viral clearance, this neuroinflammation is also a major cause of tissue damage in the CNS (16). HSV-1 induced changes of the host cell physiology can be detrimental or beneficial with regard to the clinical representation; or they can be epiphenomena.

To understand the consequences of such molecular mechanisms in the infected neurons and their surrounding cells, it is necessary to characterize such changes by open, non-hypothesis-driven, non-reductionist approaches. Transcriptome and proteome analyses of HSV-1 infected cells or tissues allow such an evaluation. Several studies have addressed the role of viral proteins and distinct host-factors by combining pull-down approaches with proteomics (17–20). Others investigated the entire proteome of non-neuronal cells such as HSV-1 infected human epithelial cells (Hep-2), human embryonic kidney cells (HEK293), human foreskin fibroblasts, and macrophages (17, 19, 21–25). However, so far there are no data on the dynamic proteome in primary cortical cultures that contain neurons and glial cells. This is of particular interest since HSV-1 does not only establish latent infections in neurons, but infects neurons and glial cells during acute CNS infections during an HSE (26–29).

Here we analyzed changes in the proteome of primary cells from the murine brain cortex upon infection with HSV-1. As different CNS-cell types may influence the host response by paracrine mechanisms, we used mixed cortical cultures that contain the major cell-types of the brain such as neurons, astrocytes, oligodendrocytes, and microglia (29). Twenty eight host proteins were differentially regulated upon HSV-1 infection including components of the inflammasome such as the NIMA-related kinase 7 (NEK7), and ArfGap1, a regulator of membrane trafficking and endocytosis which may be important for viral internalization and replication. Moreover, we evaluated the secretome relevant for paracrine signaling—an important mechanism for a coordinated tissue response upon HSV-1 infection. Seventy one proteins were enriched in the conditioned media of HSV-1 infected cortical cells including regenerative guidance cues such as semaphorin6D. These findings may lead to a better understanding of the regenerative processes after HSE and could possibly be stimulated by treatments.

## Methods

### Animals and Viruses

All experiments were approved by the Local Institutional Animal Care and Research Advisory Committee and permitted by the local authority (Lower Saxony State Office for Consumer Protection, Food Safety, and Animal Welfare Service) following the legal rules (German Animal Welfare Law, TierSchG, §4, Abs. 3) for sacrifice of animals for research purposes (institutional registry number: §4/2017/168). C57BL/6J wild-type mice were obtained from Hannover Medical School's Central Animal Facility. Animals were sacrificed between post-natal days 1–3 and used for dissection of the brain. HSV1(17+)Lox (30) and HSV1(17+)Lox-pMCMVGFP (31) stocks were propagated in BHK cells (baby hamster kidney cells, ATCC CCL-10), harvested from the medium supernatant, and titrated on Vero cells (ATCC CCL-81) as described previously (32, 33).

### Culture of Primary Cortical Cells

Whole neo-cortices from neonatal mice were digested with 25 U/mL papain, 1.65 mM L cystein (Sigma-Aldrich), 1 mM CaCl2, 0.5 mM EDTA in DMEM-GlutaMAX™ (Gibco) for 20 min at 37°C. The supernatants were discarded and inactivating solution with 2.5 mg/ml BSA (Sigma-Aldrich), 2.5 mg/ml trypsin inhibitor (Sigma-Aldrich), 10% fetal bovine serum (FBS, PAA Laboratories), 100 U/ml penicillin/streptomycin (Invitrogen), 1x MITO+ (BD Biosciences) in DMEM-GlutaMAX™ (Gibco) was added for 5 min at room temperature. Next, we replaced the supernatants with FCS-medium containing 10% FCS, 100 U/ml penicillin/streptomycin, 1x MITO+ (Corning) in DMEM-GlutaMAX™, and the tissues were sheared. After centrifugation for 5 min at 157 × g, the cell pellets were resuspended in FBS-medium. The cells were seeded in poly-L-lysine (PLL)-coated (0.5 ng/ml, Sigma-Aldrich) 75 cm^2^ cell culture flasks (proteomics) or on PLL-coated coverslips in 24-well plates pre-incubated with FCS-medium at 5% CO2 and 37°C. The cells were allowed to adhere for 30 min at 5% CO_2_ and 37°C before this medium was replaced by serum-free NBA-medium (NeurobasalA® (Gibco), B27 (Invitrogen), GlutaMAX™ (Invitrogen), 100 U/ml penicillin/streptomycin). Starvation medium was equally composed omitting B27 supplement.

### Infection

At day of *in vitro* 6 (DIV6), the primary cortical cells were incubated with CO2-independent medium (Gibco) containing 0.1% BSA for 20 min at room temperature on a rocking platform. To prepare the inoculum, HSV-1 stocks were diluted with CO2-independent medium (Gibco) containing 0.1% (w/v) BSA to a multiplicity of infection (MOI) of 10 pfu/cell (corresponding to 2.8 × 10^6^ pfu/mL), and added to the cells for 30 min on a rocking platform. After infection, cells were washed with starvation medium once and incubated with starvation medium at 37°C for 20 h.

### Proteome and Secretome Analysis

The medium supernatants were collected from 75 cm^2^ culture flasks after 20 h post infection (hpi) with HSV-1 or after a 20 h mock treatment. Cell debris was removed by filtration through Millex VV Syringe Filter Units (0.1 μm, PVDF, 33 mm; Merck Millipore). Secreted proteins were enriched by Amicon® Ultra-15 Centrifugal Filter Units with a cut-off membrane of 3 kDa (Merck Millipore). After centrifugation for 1.5–2 h at 2,400 g, the membranes were washed several times with the concentrated medium (~250 μl). For proteome analysis, the cells were washed with PBS, and incubated with Trypsin/EDTA for 5 to 10 min at 37°C. Cells were collected, centrifuged (5 min, 600 × g), and resuspended in 100 μl RIPA buffer containing 137 mM NaCl, 20 mM Tris-HCl pH 7, 525 mM β-glycerophosphate, 2 mM EDTA, 1 mM sodium-orthovanadate, 1% (w/v) sodium-desoxycholate, 1% (v/v) Triton-X-100, protease inhibitor cocktail (Roche). Cells were homogenized and lyzed with an ultrasonic homogenizer (Sonoplus HD 2070/UW 2070; Bandelin) employing 100 W s. Lysates were centrifuged (4°C, 20 min, 21,000 × g), and the supernatants containing proteins that had been solubilized from the cells were collected. The protein concentrations of both, the cell proteome (pellet lysates) and the cell secretome (filtered and concentrated media supernatants) were measured by Pierce™ BCA Protein Assay kit. Equal volumes of enriched culture supernatant (~200 μl) and equal amounts of lysate (~100 μg), were mixed with 5x Laemmli-buffer and heated for 10 min at 95°C. After incubation on ice, proteins were mixed with acrylamide 4K (40 %, AppliChem) for 30 min at room temperature for cysteine alkylation. Proteins were separated by gel electrophoresis (12.5% (w/v) polyacrylamide-gel with an amount of 1:29 of N,N'-Methylenbisacrylamid) at 100 V. Gels were stained overnight with Coomassie® Brilliant blue G250 (Merck) in 40 % methanol and 10 % acetic acid and de-stained twice with 45% methanol and 10% acetic acid for 1 h before being washed with water for several times.

### Mass Spectrometry

Gel lanes containing protein were harvested and processed for protein analyses as described previously (34). Briefly, gel pieces were de-stained with 50% acetonitrile (ACN) at 37°C and then dehydrated with 100% ACN. Residual solvent was removed in a vacuum centrifuge and an appropriate volume of a 10 ng/μL sequencing grade Trypsin (Promega) in 10% ACN, 40 mM ammoniumbicarbonate (ABC) were added. Digestion was performed over night at 37°C and was stopped by adding 100 μL of 50% ACN, 0,1% trifluoroacetic acid (TFA). Peptides were extracted using increasing concentrations of ACN, dissolved in 30 μL 2% ACN, 0.1% TFA with shaking at 800 rpm for 20 min. After centrifugation at 20,000 × g, supernatants were directly analyzed by LC-MS or stored at −20°C. Peptide samples were separated with a nano-flow ultra-high pressure liquid chromatography system (Thermo Scientific) equipped with a trapping column (3 μm C18 particle, 2 cm length, 75 μm ID, Acclaim PepMap, Thermo Scientific) and a 50 cm long separation column (2 μm C18 particle, 75 μm ID, Acclaim PepMap, Thermo Scientific). Peptides were eluted with a multi-step binary gradient: linear gradient of buffer B (80% ACN, 0.1% formic acid) in buffer A (0.1% formic acid) from 4 to 90% at a flow rate of 250 nL/min and a column temperature of 45°C. The RSLC system was directly coupled to a Nano Spray Flex Ion Soure II of an LTQ-Orbitrap Velos mass spectrometer (Thermo Scientific). Metal-coated fused-silica emitters (SilicaTip, 10 μm i.d., New Objectives) and a voltage of 1.3 kV were used for the electrospray. Overview scans were acquired at a resolution of 60 k in a mass range of m/z 300–1,600 in the orbitrap analyzer and the top 10 most intensive ions were selected for CID fragmentation in the LTQ. Dynamic exclusion was set to 70 s. Raw data were processed using Max Quant software (35) (version 1.5) and a home made data base containing human entries, common contaminants and viral proteins.

### Bioinformatics

The bioinformatics analyses were done using the Perseus software (version 1.6.0.7) (30). Each identified protein had at least three valid measurements in each experimental group (mock-infected or HSV-1 infected). Proteins that did not meet this criterion were omitted from further analysis. Missing values were sample-wise imputed by a normal distribution (down-shift 1.8 width 0.3) or by a constant value below the lowest valid measurement (in this case 13). We used the functional enrichment tool (FunRich version 3.1.3) for the gene-ontology (GO) enrichment analysis (31). The Uniprot/Swissprot rodent database was used for mapping of the proteins (release: 3rd July 2019).

### Immuno Fluorescence Microscopy

Cells were washed with PBS and fixed with 4% (w/v) paraformaldehyde (Sigma Aldrich) in PBS. Unspecific binding was blocked with 3% (v/v) normal goat serum (NGS, Gibco) and 0.3% Triton-X100 in PBS for 1 h at room temperature. The cells were incubated overnight with primary antibodies (βIII-tubulin, 1:500, Millipore; GFAP, mouse, 1:500, Sigma-Aldrich; Iba-1, 1:500, Wako Chemicals; Olig2, 1:500, Millipore) diluted in blocking solution, washed in PBS, and then treated with anti-mouse-AlexaFluor555 (1:500, goat, Molecular Probes) in blocking solution for 1 h at room-temperature. Cells were washed again with PBS, and DAPI (1:2,000, Sigma-Aldrich) was added to the second-last washing step. The cover slips were mounted on object slides with ProLong® Gold Antifade Mountant (Molecular Probes). A BX61 epifluorescence microscope equipped with CellSense dimensions software (Olympus) was used for image acquisition.

### Western Blot

Cells were washed with room temperature PBS and incubated for 15 min with ice-cold RIPA buffer [137 mM NaCl, 20 mM Tris-HCl, pH 7, 525 mM β-glycerophosphate, 2 mM EDTA, 1 mM sodium-orthovanadate, 1% (w/v) sodiumdesoxycholate, 1% (v/v) Triton-X-100, protease inhibitor cocktail (Roche)] for lysation. After sonification for 15 min, cell debris was removed via centrifugation at 4°C and 22,000 rcf for 20 min. The cleared lysates were mixed with Laemmli-buffer (80 mM Tris-HCl pH 6.8, 2% SDS (w/v), 5% (v/v) 2-mercaptoethanol and 0.01% (v/v) bromphenol blue), loaded on 12.5% polyacrylamide/SDS-gels for electrophoretic separation. After blotting on nitrocellulose membranes (GE Healthcare), following antibodies were used: self-raised polyclonal rabbit anticapsid antibody HSV-1 (32), Nek7 (Cell Signaling, monoclonal rabbit, #3057), and histone H3 (Cell Signaling, polyclonal rabbit, #9715). Densitometric values of Nek7 protein bands of a biological independent replicate were normalized by their geometric mean and subsequently by the corresponding and geometric mean normalized H3 bands.

## Results

### HSV-1 Infects Primary Cortical Brain Cells

**Figure 1:**
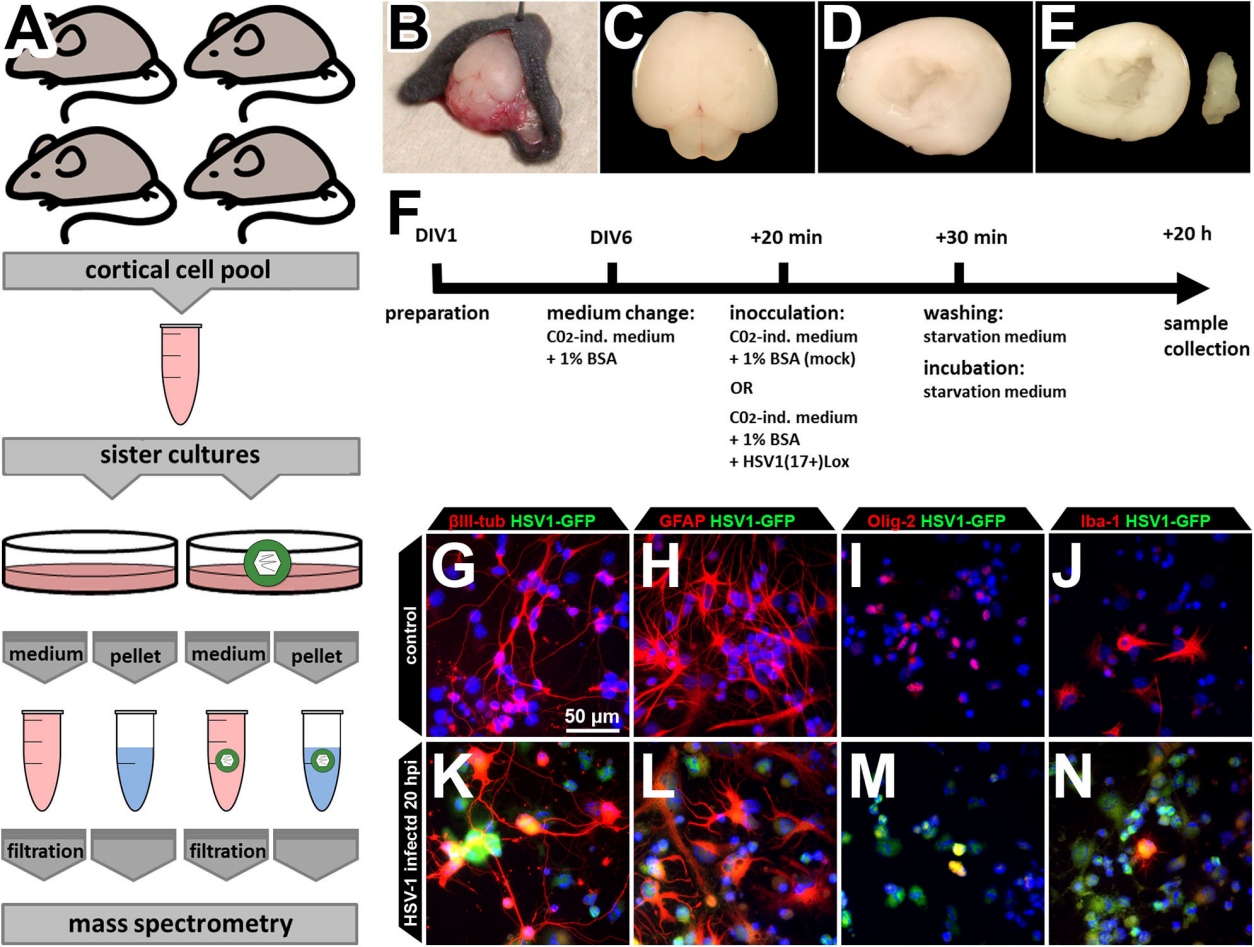
Experimental design. **(A)** The cells of four mice were pooled for a single HSV-1 or mock control infection in sister cultures. This procedure was repeated three times to obtain three biological independent replicates. Cortices of neonatal mouse brains were dissected **(B–F)**, pooled, cultured, and infected **(F)** before samples were collected. Medium supernatant as well as cell pellets were used for subsequent mass spectrometry analysis. **(B–E)** Neonatal C57B6/J-mice were used for preparation of the brain **(B,C)**. Cortex was dissected **(D)** and hippocampus removed thereby obtaining the neocortex **(E)**. Neocortical cells were pooled before plating, assuring an identical cell composition **(A)**. **(F)** The cells were kept in culture beginning from day of *in vitro* 1 (DIV1). At DIV6, medium was changed to CO_2_-independent medium for 20 min. The same medium served as inoculation medium either containing HSV1(17+)Lox in a multiplicity of infection (MOI) equivalent to 10 (corresponding to 2.8 × 10^6^ pfu/ml) or no virus as the mock-infected control. Addition of the virus was defined as 0 h post infection (hpi). After 30 min, cells were washed and incubated to 20 hpi with starvation medium devoid of any growth factors or serum. **(G–N)** The culture comprises the major CNS cell types identified by the expression of different marker proteins such as βIII-tubulin (neurons), glial fibrillary acidic protein (GFAP, astrocytes), oligodendrocyte transcription factor (Olig-2, oligodendrocytes), and ionized calcium-binding adapter molecule 1 (Iba-1, microglia). After incubation with a HSV-1(17+)LoxGFP at a MOI = 10 all of those cell types became infected **(K–N)**.

HSV-1 infects neurons, astrocytes, and oligodendrocytes in HSE-patients, mice, and human and murine primary cortical cultures (26–29, 33). Moreover, HSV-1 infection modulates the physiology of other cell types such as oligodendrocytes and microglia, the resident “macrophages” of the brain (27). To address the possible involvement of different CNS cell types as well as their interaction, we developed a protocol to investigate the proteome and the secretome of mixed primary cultures from the brain cortices of neonatal C57BL/6J mice (Figure 1A). As previously established and characterized (29), we dissected the cortices, and cultured the cells for six days to obtain an *ex vivo* system which contains the major CNS-cell types (Figures 1B–J). The cells were inoculated with HSV-1 for 20 h prior to processing the samples for cell proteome analyses. This time window allows protein regulation and secretion, but prevents HSV-1 induced cell death (29). HSV-1 preferentially infected astrocytes, followed by neurons, and oligodendrocytes while there were only a few infected microglia (Figures 1K–N) as reported before (29). Importantly, we observed no gross detachment of primary cells upon HSV-1 infection (Figures 1G–N). The conditioned media containing secreted proteins and vesicles were processed for protein secretome analyses.

### HSV-1 Infection Significantly Changes 28 Proteins in Mixed Cortical Cell Cultures

**Figure 2:**
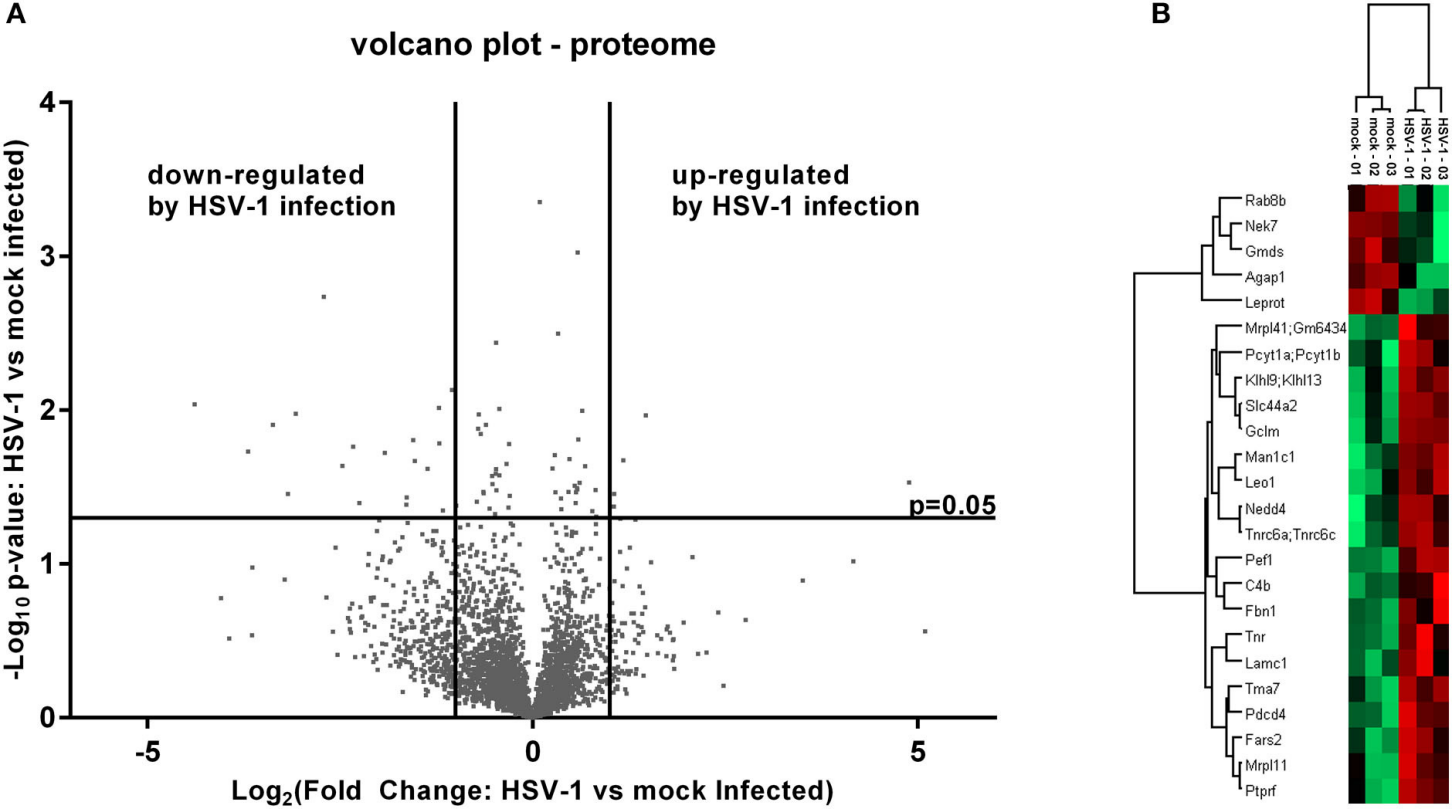
Mass spectrometry analysis of the pelleted cells—proteome. **(A)** Volcano plot of label free quantification (LFQ) values used for the calculation of a fold change and a p-value (*n* = 3). A fold change > 2 and a p-value smaller than 0.05 were used for the identification of regulated proteins either as being down- or up-regulated. **(B)** Significantly altered proteins were subjected to a hierarchical clustering analysis with low LFQ-values in red and high LFQ-values in green.

The cell extracts were analyzed to evaluate the host proteome response to infection. We prepared samples in triplicates and used label free quantification (LFQ) of protein groups to estimate the fold changes for HSV-1 vs. mock infected cells (36). There was a low proportion of imputed values, and multi-scatter plots of label free quantification (LFQ) values showed a good correlation between each of the samples: the Pearson coefficients ranged from 0.722 to 0.96 confirming the data quality (Figure S1). The ratios of protein LFQ-intensities from HSV-1 infected vs. mock treated cells (fold change), and the corresponding p-values were displayed in volcano plots to highlight significant changes in protein composition. Both, the fold changes as well as p-values served as criteria to indicate a relevant change: Fold changes had to be >2, and the *p*-value thresholds equal to or below 0.05 (Figure 2A).

**Figure 3:**
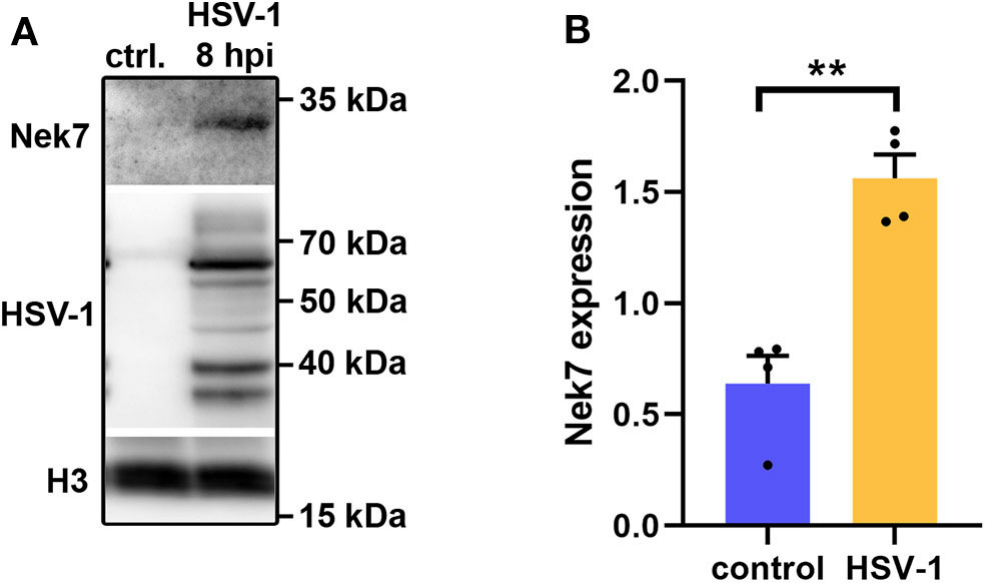
Nek7 Western blots of HSV-1 infected primary cortical cells. Primary cortical cells were infected at DIV6 with HSV-1(17+)LoxGFP at a MOI = 10 and analyzed 8 hpi. **(A)** Representative Western blot of mock infected control (ctrl.) cells and HSV-1 infected cells developed with a Nek7, HSV-1, and histone H3 antibody. **(B)** Densitometric analysis of Nek7 expression normalized by histone H3 expression. Bar graph displays mean + SEM and individual datapoints. *n* = 4, Student's t-test with **p < 0.01.

Five proteins were significantly up-regulated and 23 down-regulated upon HSV-1 infection. A hierarchical clustering analysis demonstrated consistent replicate samples within one group, and difference among the groups (HSV-1 vs. mock-treated) (Figure 2B). Three proteins were down-regulated by more than 10 fold including the translation machinery associated 7 homolog (Tma7), tenascin-R (Tnr), and programmed cell death protein 4 (Pdcd4) (Table 1). The NIMA-related kinase 7 (NEK7) LFQs displayed a strong, almost 30 fold upregulation 20 hpi. To test for an early Nek7 upregulation, primary cortical cells were infected and evaluated 8 hpi (Figure 3). Western blots confirmed the Nek7 up-regulation at an earlier time point after infection indicating a rapid reaction of the cells due to HSV-1 infection (Figures 3A,B).

**Table 1:** Fold changes of proteins in primary cortical cells upon HSV-1 infection.

**Gene name**	**Protein IDs**	**Fold Change^**a**^**	***p*^**b**^**
NEK7	Q9ES74	29.5	0.029
Leprot	O89013	2.8	0.011
Agap1	Q8BXK8	2.3	0.021
Rab8b	P61028	2.1	0.035
Gmds	Q8K0C9	2.1	0.042
Gclm	O09172	−2.1	0.007
Pcyt1a;Pcyt1b	P49586;Q811Q9	−2.2	0.045
Klhl9;Klhl13	Q6ZPT1;Q80TF4	−2.3	0.016
Man1c1	Q6NXK9	−2.3	0.010
Mrpl41;Gm6434	Q9CQN7;F7CXS7	−2.6	0.024
Ptprf^3^	A2A8L5	−2.9	0.021
Tnrc6a;Tnrc6c^c^	Q3UHK8;Q3UHC0	−2.9	0.016
Lamc1^c^	F8VQJ3;P02468	−3.1	0.037
Nedd4	P46935	−3.1	0.041
Leo1	Q5XJE5	−3.8	0.019
Mrpl11^c^	Q9CQF0	−4.8	0.040
Fars2^c^	Q99M01	−5.0	0.017
Fbn1	A2AQ53	−5.5	0.023
Pef1^c.d^	Q8BFY6	−6.6	0.002
Slc44a2^c^	Q8BY89	−8.4	0.011
C4b^c.d^	P01029	−9.1	0.035
Tma7	Q8K003	−10.4	0.012
Tnr	Q8BYI9	−12.9	0.019
Pdcd4^c^	Q61823	−21.0	0.009

*Fold Change^a^ (HSV-1 infected over mock-infected) was calculated from the mean intensities in one group and is presented here with their corresponding p-value^b^. Nine of the down-regulated targets were identified by a proteomic study of HSV- infected primary human foreskin fibroblasts (24)^c^. Pef 1 and C4b are down-regulated in our cortical brain cultures while they were up-regulated in fibroblasts (24)^d^*.

### HSV-1 Infection Modulates Expression of Proteins Involved in Inflammatory Cell Death and Organelle Transport

**Figure 4:**
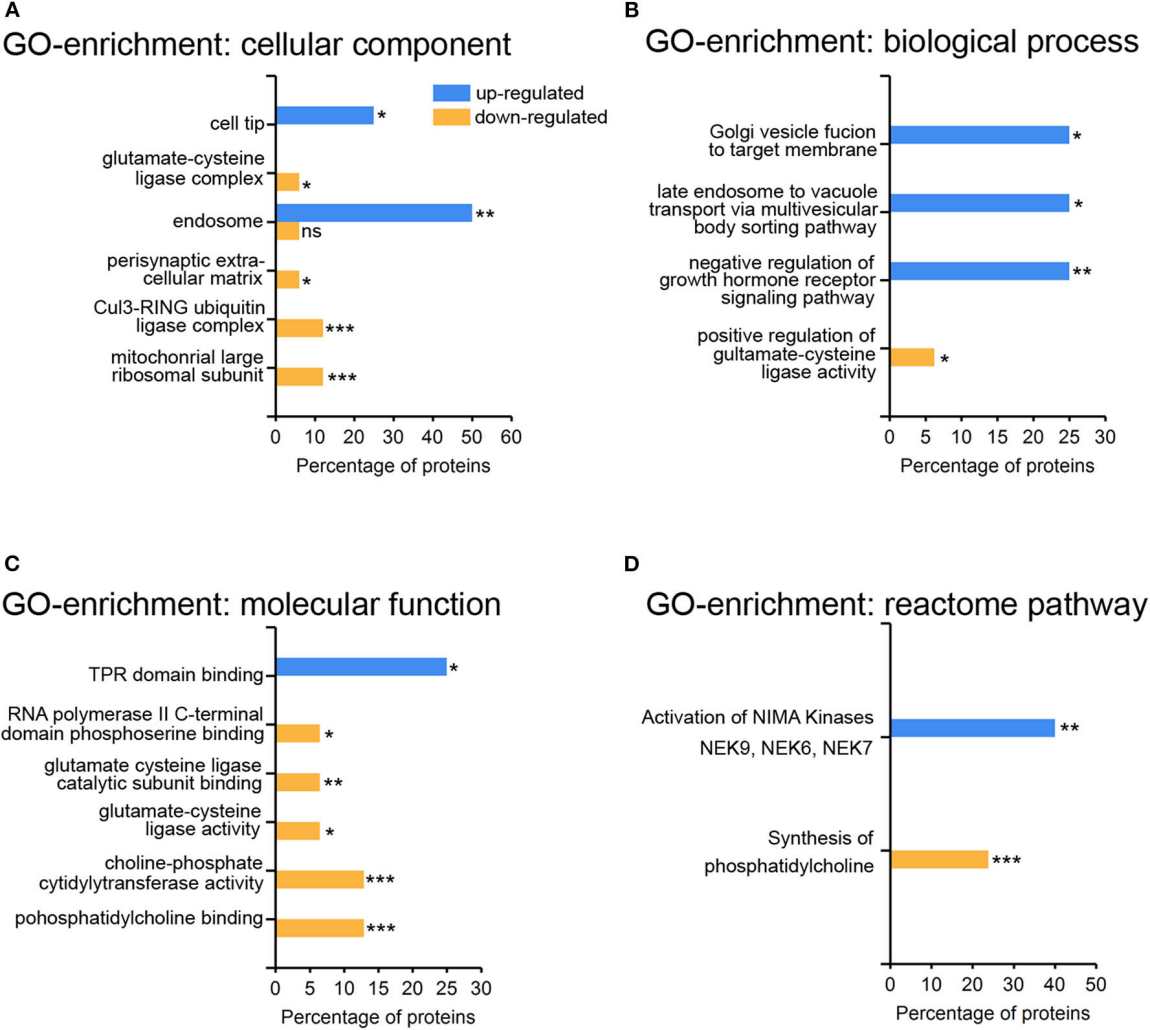
Gene-ontology enrichment of regulated proteins in primary cortical cells upon HSV-1 infection. Proteins from Table 1 were subjected to the Functional enrichment tool (FunRich v 3.1.3) with the Uniprot rodent database (April 2019) and the BioGRID interaction Database (3.5.171, March 2019). A total number of 28 proteins could be mapped with five up- and 23 down-regulated proteins. Gene-ontology (GO) enrichment analyses were performed by the categories cellular component **(A)**, biological process **(B)**, molecular function **(C)**, and reactome pathways **(D)** with the percentage of the HSV1-regulated proteins having a particular enrichment-tag *p < 0.05, **p < 0.01, ***p < 0.001.

The abundance of specific proteins differs in response to viral infection: restricting proteins are up-regulated to mount an anti-viral response, and enabling proteins to foster viral replication and assembly. The HSV-1 host shutoff response leads to a down regulation of proteins to suppress innate immunity and to stop synthesis of proteins not required for infection (37). The higher proportion of down-regulated rather than up-regulated proteins may be due to the action of the HSV-1 virion host shutoff (vhs) protein and to global transcriptional read-through which inhibits host translation (38, 39). We performed a gene-ontology (GO)-enrichment analyses for the up-regulated and the down-regulated proteins separately (Figure 4). Gene-ontology terms for endosomes and intracellular membrane trafficking were significantly enriched among the set of up-regulated proteins (Figures 4A,B). This may reflect the role of endocytosis and the secretory pathway for HSV-1 infection (40–42). Interestingly, just one gene-ontology term was enriched within the up-regulated set of proteins—the NIMA kinases (Figure 4D) which regulate innate immunity. NIMA kinases, such as NEK7, are crucial components of the inflammasome, a multi-protein complex that regulates a specific form of programmed cell death, the inflammatory pyroptotic cell death (43, 44). Among the down-regulated proteins, there was an enrichment for proteins participating in phosphatidylcholine metabolism—the major phospholipid component of plasma membranes (Figures 4C,D). HSV-1 interferes with this pathway in the context of viral envelopment (45), and activates the phospholipid scramblase to promote cell entry (46). Moreover, proteins modulating the glutamate cysteine-ligase (GCL) activity were enriched in the set down-regulated proteins (Figures 4B,C). GCL regulates glutathione levels, which are critical for protective, anti-viral responses in microglia (47, 48).

### The Secretome of HSV-1 Infected Cortical Cells

**Figure 5:**
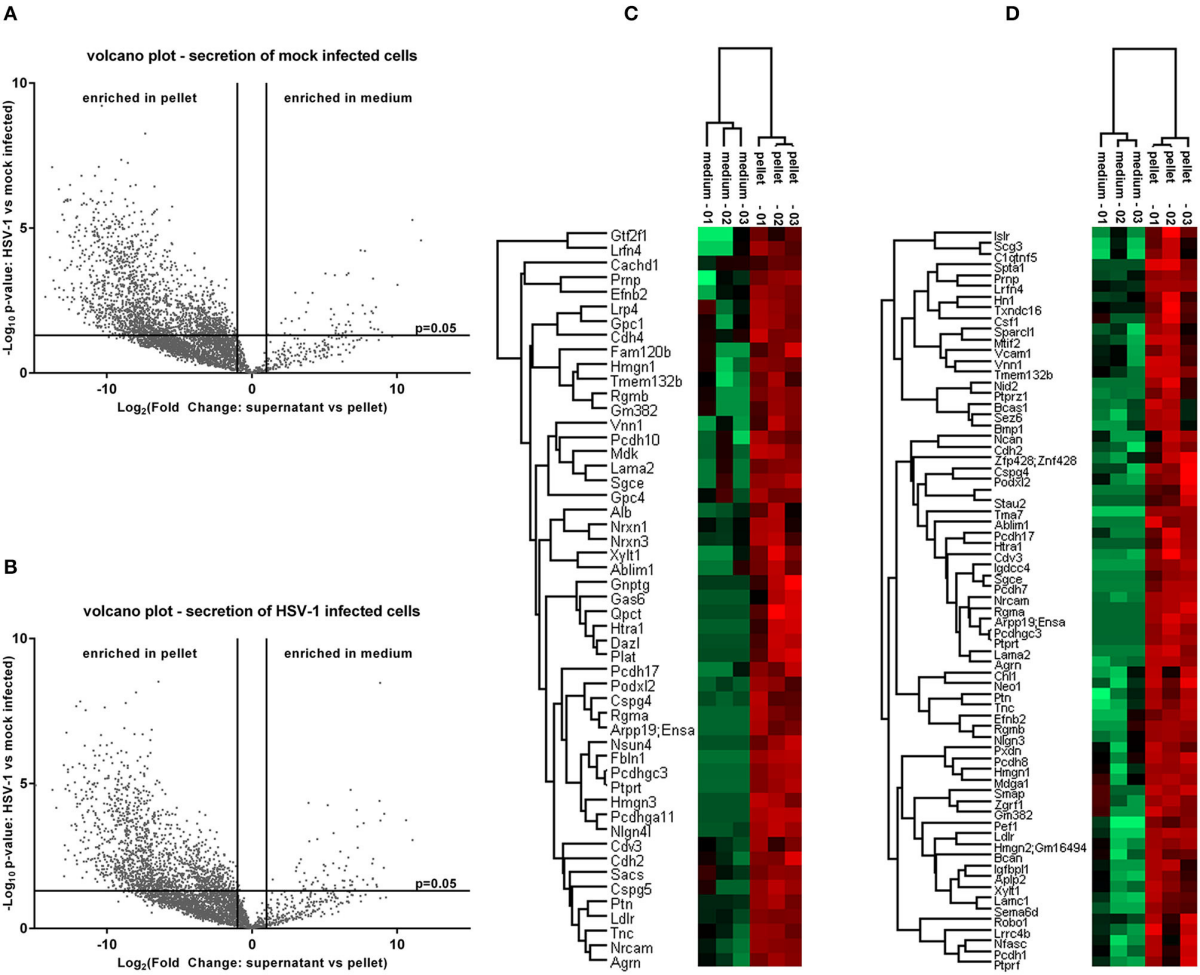
Proteome analysis of conditioned medium compared to pelleted cells. The raw intensities of all replicates were used to calculate a fold change between the conditioned medium and the pellet as well as the corresponding p-values. Secreted proteins which are enriched in the conditioned medium emerge in the upper right of a volcano plot for control-conditions **(A)** or HSV-1 infected primary cortical cultures **(B)**. They were identified by a fold change >2 and a p-value lower than 0.05. A hierarchical clustering was performed with the medium-enriched proteins of mock-infected control cells **(C)** or HSV-1 infected cells **(D)**. The heat-maps give highly abundant proteins in green and low expressed proteins in red.

**Figure 6:**
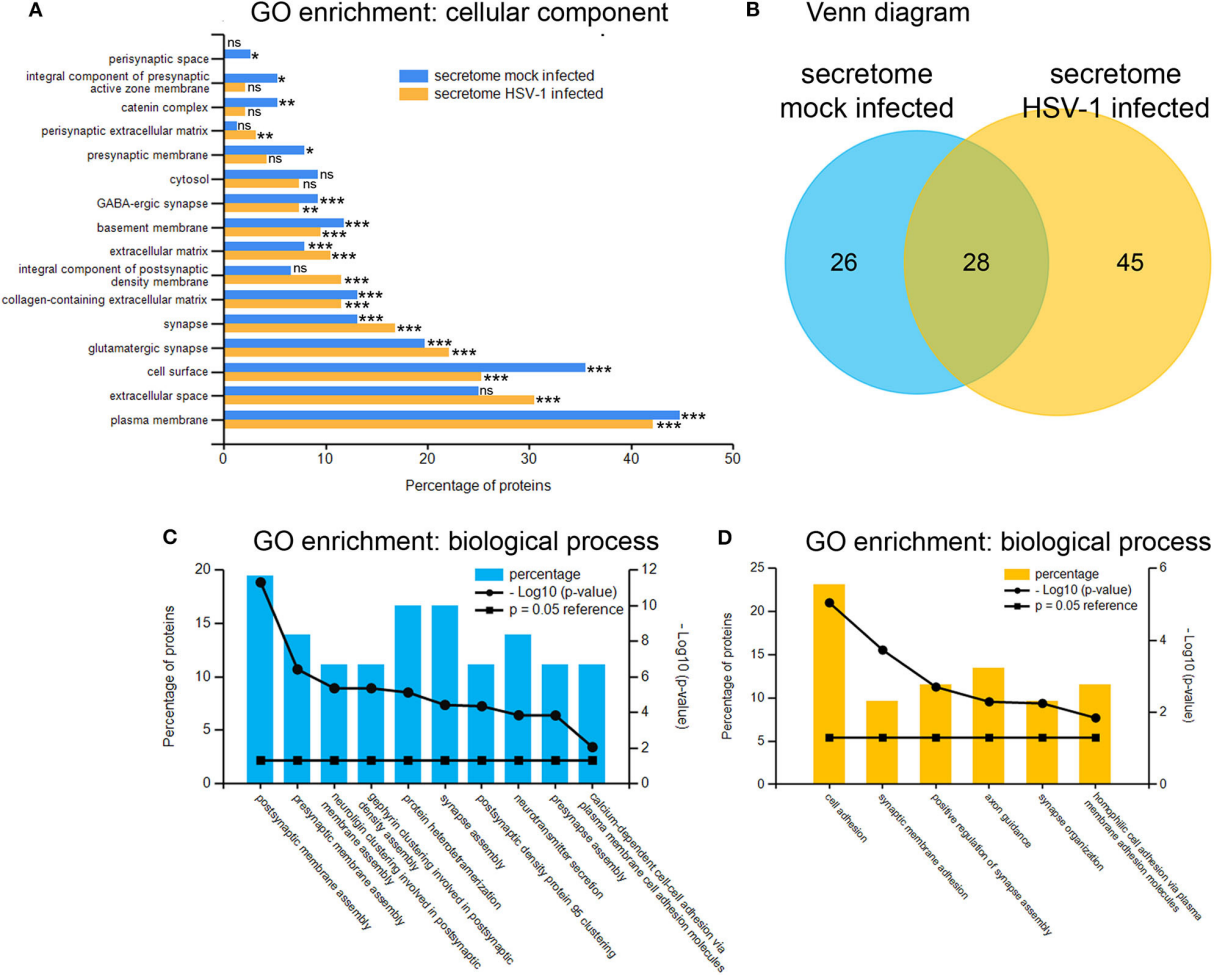
Gene ontology enrichment of proteins enriched in conditioned medium. GO-analysis by the category of cellular component of proteins from the mock-infected (blue) as well as the HSV-1 infected secretome mostly revealed extracellular localization-tags **(A)**. A total number of 54 proteins were putatively secreted by mock-infected control-cells and 73 proteins by HSV-1 infected cells with an overlap of 28 proteins **(B)**. The 26 mock-infected-only **(C)** and the 45 HSV-infected-only **(D)** secretory proteins were analyzed for GO-enrichments in the category biological process with *p < 0.05, **p < 0.01 and ***p < 0.001.

Next, we analyzed the secretion-profile of HSV-1 vs. mock-treated cortical cells. First, we identified the secreted proteins and compared their abundance in the conditioned media to that of the cells. We consider proteins enriched in the conditioned medium to be specifically released from infected cells. The low rate of imputed values and good Pearson coefficients indicated a good quality of the data (Figure S2). Not surprisingly, most proteins were more abundant in the cell fractions than in the corresponding media (Figures 5A,B). However, 54 proteins were significantly enriched in the conditioned media of mock-infected cells (Figure 5A) while this number was 73 for HSV-infected cells (Figure 5B). A hierarchical clustering analysis separated these two experimental conditions. Single samples show a consistent pattern between HSV-1 infected and mock-treated cells for all proteins in both analyses (Figures 5C,D). These secretomes contain proteins implicated in paracrine signaling as well as constituents of extracellular vesicles (EV) such as exosomes and microvesicles (49). Thus, we analyzed the secretome of HSV-1 and mock-treated cells for GO-enrichment in the cellular component category (Figure 6A). More than 40 % of the proteins were plasma-membrane proteins, 25–30% secreted proteins, and only 10% were cytosolic proteins. Importantly, the latter were not enriched, and there was no difference between HSV1- and mock-treated cells indicating only a minor contamination by cellular debris (Figure 6A). Twenty eight proteins were present in both secretome sets. The conditioned media of mock-infected cortical cells contained 26 specific proteins, and there were 45 proteins specifically secreted from cells infected with HSV-1 (Figure 6B). A GO-analysis of those subsets revealed a common role in synaptic processes. Interestingly, the secretome of HSV-1 infected cells exclusively showed GO-enrichment of positive regulation of axonal guidance (Figures 6C,D). Proteins involved in axonal guidance include neogenin1, Robo1, Close homolog of L1, neurofascin, and semaphorin6D, which indicates a regulatory potential of the host for neuronal plasticity processes upon infection.

## Discussion

To obtain further insights into the physiological changes upon an HSV-1 infection of the brain during HSE, we analyzed the proteome and secretome of HSV-1 infected murine, primary cortical brain cells by mass spectrometry. HSV-1 preferentially infects neurons, astrocytes and oligodendrocytes but not microglia in the brain (26–28, 33, 50) and has a similar cell tropism in our primary murine cortical culture (29). In this mixed culture, the specific cell types react differently to HSV-1 infection: strong responses in a large population will become apparent in the cell proteomes, and particularly in the secretome while a moderate, yet specific modulation of few cells will not become manifest in the secretome.

In contrast, homogenous cultures of one cell type allow detection of a higher number of proteins. Pioneering quantitative mass spectrometry studies have identified a broad range of nuclear and cytoplasmic proteins whose abundance changes upon HSV-1 infection, e.g., in human HEp-2 cells (21) and HEK293 cells (22). Detailed analyses of human foreskin fibroblasts identified almost 60 proteins, involved in immune defense responses, extracellular matrix organization, and DNA damage that are down-regulated during the course of an HSV-1 infection (23). A further recent in-depth analysis of HSV-1 infected human fibroblasts identified 34 upregulated and almost 500 down-regulated host proteins (24). In our analysis of primary murine cortical brain cells we confirmed 9 of the 496 down-regulated proteins from the human fibroblasts. One of those is the programmed cell death protein 4 (Pdcd4) with a 20 fold decreased expression in our dataset. Pdcd4 is a direct target of the HSV-1 U_S_3 protein kinase thereby protecting host cells from apoptosis (51).

In cortical brain cells, HSV-1 infection prominently induced the expression of proteins that regulate membrane homeostasis and trafficking including endosomes and the Golgi apparatus. While HSV-1 enters neurons and possibly other cell types by direct fusion of viral envelopes with the plasma membrane, many cell types are infected via endocytosis (40). In particular, HSV-1 internalization by macropinocytosis requires a major re-arrangement of the actin cytoskeleton to engulf the virions by plasma membrane extensions (42, 52). Interestingly, HSV-1 infection of cortical cells induced a prominent up-regulation of ArfGap1 which is critically involved in the regulation of membrane trafficking and macropinocytosis. ArfGap1 is a sensor for membrane tension, becomes activated by membrane curvature and regulates trafficking via inactivation of Arf1 (53). Moreover, ArfGap1 stabilizes cortical F-actin thereby hindering the uptake of *mycobacterium tuberculosis* to endothelial cells thus being a host restriction factor for pathogen uptake (54). However, it is yet unresolved whether or not the HSV-1 induced up-regulation of ArfGap1 protects CNS cells from viral entry and trafficking. Moreover, endosomes and the Golgi apparatus play an important role in viral trafficking and later stages of viral assembly such as capsid envelopment. Rab GTPases critically regulate the transport of plasma membrane components to recycling endosomes ready to envelope the capsid (55–57). Moreover, some Rab GTPases become incorporated into mature HSV-1 particles where they are important for HSV-1 replication (58). Interestingly, Rab8b was up-regulated in cortical brain cells suggesting a role of this GTPase in HSV-1 life-cycle. While there are no reports about a role of Rab8b in HSV-1 so far, West Nile virus production critically depends on this GTPase (59).

Proteins which stimulate glutamate cysteine ligase (GCL) activity were down regulated in HSV-1 infected cortical brain cells. Since GCL controls the glutathione level, lowered glutathione levels are expected in primary cortical neurons. Glutathione is critical for HSV-1 production. In fact, HSV-1 needs to lower cellular glutathione levels for an efficient viral replication and GCL supplementation protects mice from HSV-1 infections (48, 60, 61). In primary cortical cultures, the virus was mainly detected in neurons, astrocytes and oligodendrocytes. Only a few microglia were cultured with a negligible fraction of infected cells (29). Interestingly, higher glutathione levels protect microglia from HSV-1 infections which could explain the low abundance of HSV-1 positive microglia (47). HSV-1 interferes with a number of intrinsic and innate resistance mechanisms (62). An efficient viral replication relies for example on the inactivation of the pattern recognition receptor AIM2 via tegument protein VP22. As a consequence AIM2 is not able to activate the inflammasome (63). The inflammasome is a cytosolic protein complex which cleaves pro-protein forms of inflammatory cytokines such as IL-1beta and IL-18. This pro-inflammatory effect is often combined with a pyroptotic cell death (43). NEK7 is a central component of the inflammasome (44). Interestingly, NEK7 was up-regulated already at 8 hours post infection and almost 30 fold up-regulated 20 hpi. This could be a rapid and potential compensatory reaction of the host-cells. However, the consequences of this up-regulation remain elusive. An anti-viral inflammation may be beneficial preventing further spread. On the other hand, neuroinflammation may be detrimental hindering neuroregeneration (16).

We detected a significant number of membrane proteins in the secretome of HSV-1 infected cortical cells, indicating an abundance of extracellular vesicles in the secretome. HSV-1 infection indeed, elicits the evasion of specific microvesicles from an oligodendrocyte cell line which contain virions and are able to infect neighboring cells (64). Another population of extracellular vesicles from HSV-1 infected cells contributes to the propagation of innate immunity signaling among infected and non-infected neighboring cells. Those vesicles contain DNA-sensing molecules such as STING and block virus replication (65). Interestingly, we found the High-Mobility Group Nucleosome-Binding Protein 1 (HMGN1) in the HSV-1 secretome, which is a known component of exosomes (66) and and a modulator of innate immunity via TLR signaling (67). HSV-1 infected cells also secreted proteins which regulate axonal guidance—an important process in regenerating neurons. Those included neogenin1, Robo1, close homolog of L1, neurofascin, and semaphorin6D. Little is known about the role of axonal guidance molecules in HSV-1 infections of the brain. However, the expression of corneal semaphorin6D increases after HSV-1 infections and this is necessary for an axonal regeneration after virus induced denervation (68). Therefore, our secretome data indicate an early induction of repair mechanism in infected cortical cells. These mechanisms may be important links for therapeutic strategies which trigger regeneration after HSV-1 infections of the brain.

## Data Availability Statement

The mass spectrometry proteomics data have been deposited to the ProteomeXchange Consortium via the PRIDE (69) partner repository with the dataset identifier PXD018819.

## Ethics Statement

The animal studies were reviewed and approved by the Local Institutional Animal Care and Research Advisory Committee and permitted by the local authority (Lower Saxony State Office for Consumer Protection, Food Safety, and Animal Welfare Service) following the legal rules (German Animal Welfare Law, TierSchG, §4, Abs. 3) for sacrifice of animals for research purposes (institutional registry number: 4/2017/168).

## Author Contributions

PC conceptualized the project. VR conducted the experiments with the support of AB and AP. NH and AP analyzed the mass spectrometry data. NH performed the bioinformatics analyses. NH, BS, AP, and PC interpreted the data and wrote the manuscript. All authors contributed to the article and approved the submitted version.

## Conflict of Interest

The authors declare that the research was conducted in the absence of any commercial or financial relationships that could be construed as a potential conflict of interest.
