# Supplementary material for: The Proteome and Secretome of Cortical Brain Cells Infected With Herpes Simplex Virus
Source: Front Neurol. 2020 Aug 27;11:844. doi: 10.3389/fneur.2020.00844 (PMC7481480; doi:10.3389/fneur.2020.00844)
Supplement: Supplementary file 1 [file Data_Sheet_1.docx]

Supplementary Material


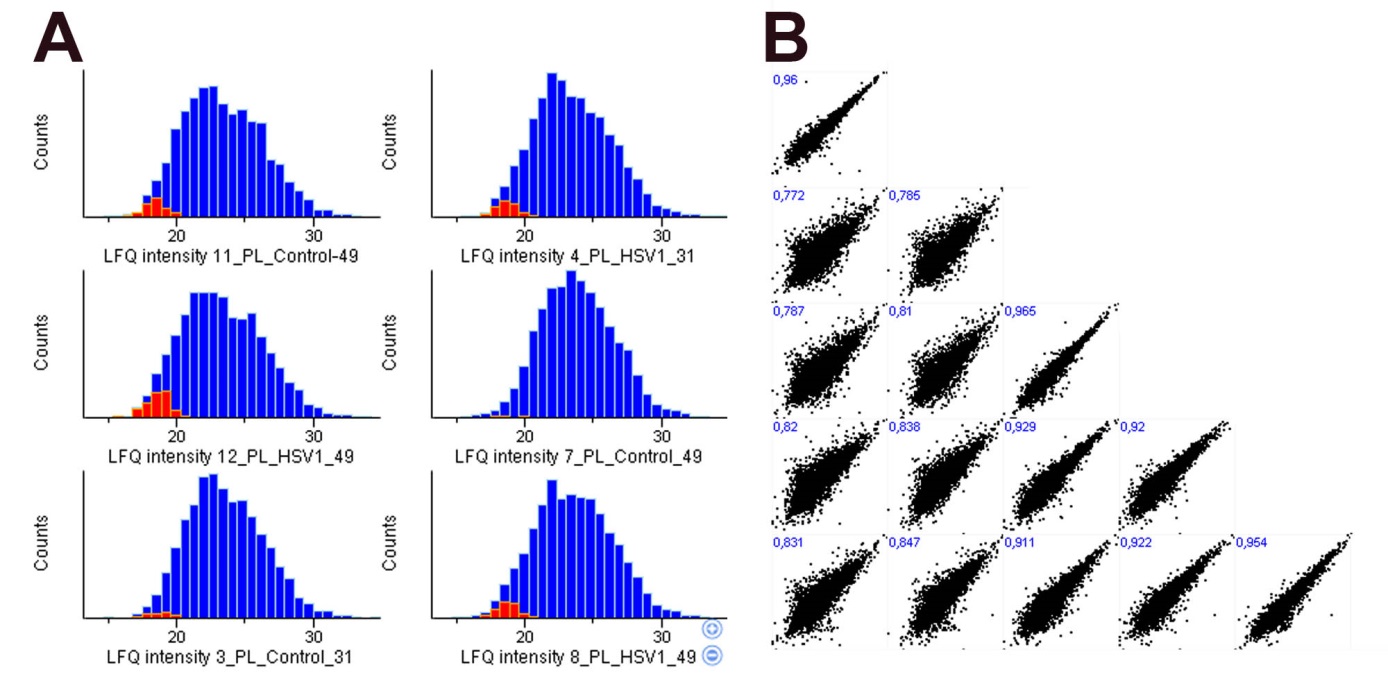


**Figure S1: Data and imputation quality in cell pellet samples.** Histograms of the LFQ-intensities (blue) as well as imputed values (red) of quantified proteins from the different cell pellet samples **(A)**. Multi-scatter plot with pearsons correlation coefficient for LFQ-values of the pellet samples **(B)**.


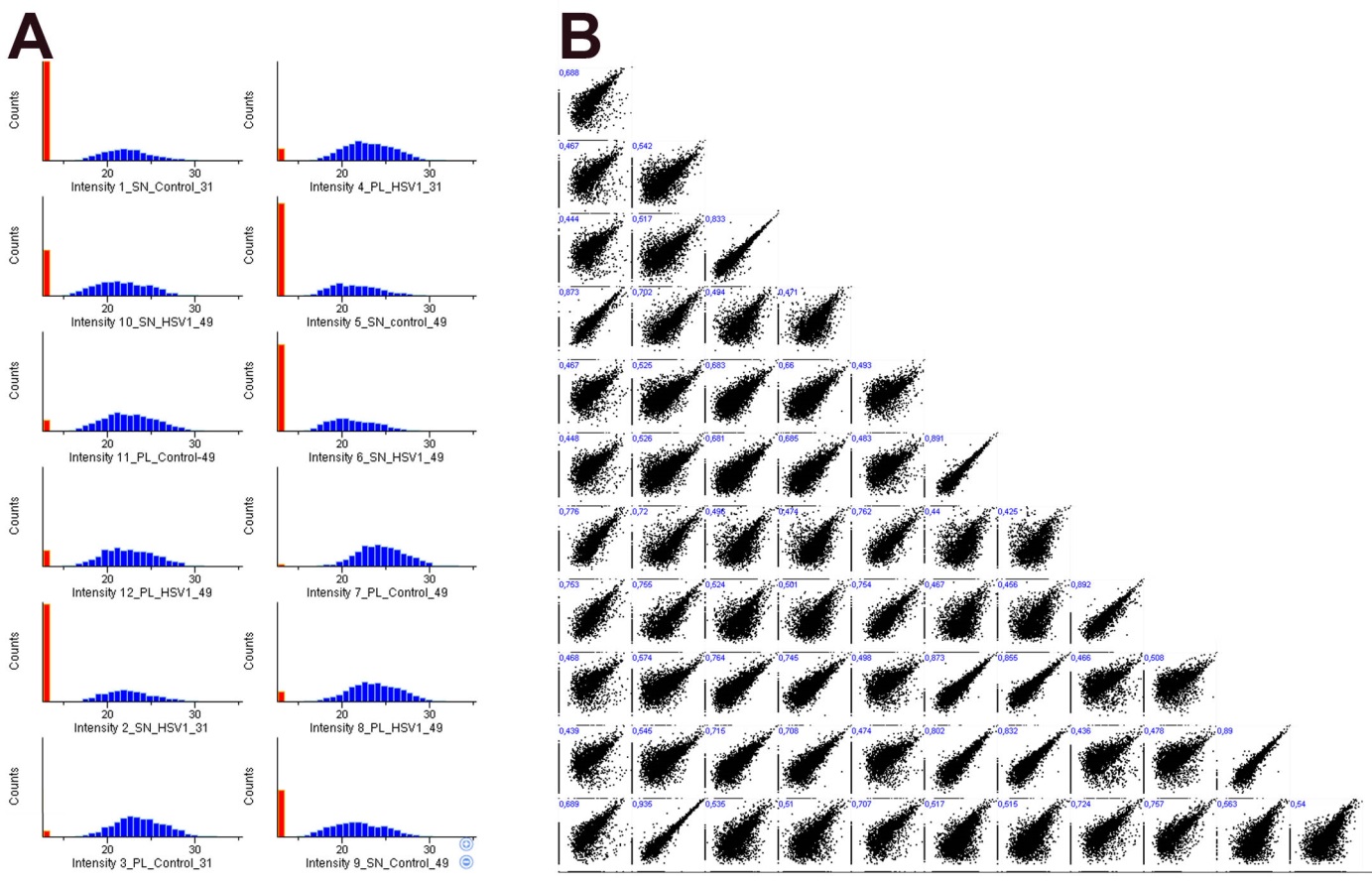


**Figure S2: Data and imputation quality in cell pellet and supernatant samples.** Histograms of the raw-intensities (blue) as well as imputed values (red) for the different cell pellet samples and the medium-supernatant samples **(A)**. Multi-scatter plot with pearsons correlation coefficient for raw-intensity values **(B)**.

**Table S1**

| Gene name | protein IDs | Control  Fold Change | Control  p-value | HSV-1  Fold Change | HSV-1  p-value |
| --- | --- | --- | --- | --- | --- |
| Pcdhgc3 | Q91XX1 | 3211 | 3,E-05 |  |  |
| Fbln1 | Q08879 | 2135 | 5,E-06 |  |  |
| Nsun4 | Q9CZ57-2 | 1048 | 9,E-04 |  |  |
| Fam120b | Q6RI63;Q6RI63-2 | 501 | 4,E-02 |  |  |
| Ablim1 | Q8K4G5 | 414 | 3,E-02 |  |  |
| Pcdh17 | E9PXF0 | 375 | 5,E-03 |  |  |
| Pcdhga11;Pcdhga3;Pcdhga8 | Q91XY8;Q91XY5;  Q91XY0 | 375 | 2,E-06 |  |  |
| Gm382 | B1AXN3 | 345 | 3,E-02 |  |  |
| Rgma | Q6PCX7 | 325 | 6,E-04 |  |  |
| Cspg5 | Q71M36 | 323 | 9,E-03 |  |  |
| Qpct | Q9CYK2 | 303 | 7,E-03 |  |  |
| Gas6 | Q61592 | 293 | 3,E-02 |  |  |
| Vnn1 | Q9Z0K8 | 284 | 2,E-02 |  |  |
| Nlgn4l | B0F2B4 | 221 | 6,E-05 |  |  |
| Htra1 | Q9R118 | 221 | 9,E-03 |  |  |
| Dazl | Q64368 | 198 | 5,E-03 |  |  |
| Ptprt | Q99M80 | 179 | 6,E-05 |  |  |
| Xylt1 | Q811B1 | 125 | 9,E-03 |  |  |
| Hmgn1 | P18608;D3Z607 | 117 | 4,E-02 |  |  |
| Gnptg | Q6S5C2 | 109 | 2,E-02 |  |  |
| Rgmb | Q7TQ33 | 101 | 2,E-02 |  |  |
| Hmgn3 | Q9DCB1 | 101 | 4,E-04 |  |  |
| Plat | P11214 | 97 | 4,E-03 |  |  |
| Arpp19;Ensa | P56212 | 92 | 4,E-04 |  |  |
| Lama2 | Q60675 | 89 | 2,E-02 |  |  |
| Gpc1 | Q9QZF2 | 88 | 4,E-02 |  |  |
| Pcdh10 | Q925I8 | 87 | 4,E-02 |  |  |
| Prnp | P04925 | 77 | 4,E-02 |  |  |
| Efnb2 | P52800 | 64 | 2,E-02 |  |  |
| Lrp4 | Q8VI56 | 62 | 5,E-02 |  |  |
| Lrfn4 | Q80XU8 | 57 | 3,E-02 |  |  |
| Alb | P07724 | 55 | 3,E-02 |  |  |
| Gtf2f1 | Q3THK3 | 54 | 4,E-02 |  |  |
| Ptn | P63089 | 52 | 4,E-04 |  |  |
| Mdk | P12025 | 50 | 1,E-02 |  |  |
| Sgce | O70258 | 47 | 2,E-02 |  |  |
| Agrn | A2ASQ1 | 44 | 6,E-03 |  |  |
| Sacs | Q9JLC8 | 43 | 1,E-02 |  |  |
| Ldlr | P35951 | 33 | 4,E-04 |  |  |
| Nrcam | Q810U4 | 18 | 2,E-03 |  |  |
| Cspg4 | Q8VHY0 | 15 | 4,E-03 |  |  |
| Tnc | Q80YX1 | 13 | 8,E-03 |  |  |
| Tmem132b | F7BAB2 | 12 | 3,E-02 |  |  |
| Nrxn3 | Q6P9K9 | 9 | 1,E-02 |  |  |
| Podxl2 | F6TE23 | 8 | 2,E-03 |  |  |
| Cdh2 | P15116 | 7 | 2,E-02 |  |  |
| Nrxn1 | Q9CS84 | 6 | 5,E-02 |  |  |
| Cdh4 | P39038;Q80ZV4 | 5 | 1,E-02 |  |  |
| Gpc4 | P51655 | 5 | 4,E-02 |  |  |
| Cdv3 | Q4VAA2 | 4 | 2,E-02 |  |  |
| Cachd1 | Q6PDJ1 | 2 | 5,E-02 |  |  |
| Ablim1 | Q8K4G5 |  |  | 2124 | 9,E-04 |
| Pcdhgc3 | Q91XX1 |  |  | 1567 | 2,E-04 |
| Rgma | Q6PCX7 |  |  | 551 | 1,E-04 |
| Nid2 | O88322 |  |  | 538 | 8,E-03 |
| Lama2 | Q60675 |  |  | 461 | 2,E-04 |
| Tma7 | Q8K003 |  |  | 453 | 3,E-09 |
| Igdcc4 | Q9EQS9 |  |  | 441 | 4,E-05 |
| Gm382 | B1AXN3 |  |  | 389 | 4,E-02 |
| Scg3 | P47867 |  |  | 380 | 3,E-02 |
| Ldlr | P35951 |  |  | 325 | 4,E-03 |
| Igfbpl1 | Q80W15 |  |  | 321 | 2,E-02 |
| Islr | Q6GU68 |  |  | 318 | 2,E-02 |
| Pcdh17 | E9PXF0 |  |  | 310 | 6,E-03 |
| Sparcl1 | P70663 |  |  | 246 | 3,E-02 |
| Pcdh7 | E9Q2S0 |  |  | 206 | 2,E-04 |
| Ptn | P63089 |  |  | 202 | 2,E-02 |
| Spta1 | P08032 |  |  | 155 | 2,E-03 |
| Agrn | A2ASQ1 |  |  | 154 | 3,E-04 |
| Pcdh8 | Q7TSK3 |  |  | 151 | 2,E-02 |
| Arpp19;Ensa | P56212 |  |  | 148 | 8,E-05 |
| C1qtnf5 | F8WHS3;Q8K479 |  |  | 136 | 4,E-02 |
| Hmgn1 | P18608;D3Z607 |  |  | 120 | 2,E-02 |
| Vnn1 | Q9Z0K8 |  |  | 118 | 3,E-02 |
| Ptprt | Q99M80 |  |  | 110 | 2,E-05 |
| Bmp1 | P98063 |  |  | 109 | 3,E-02 |
| Efnb2 | P52800 |  |  | 108 | 2,E-02 |
| Htra1 | Q9R118 |  |  | 99 | 4,E-03 |
| Nlgn3 | A2AGI2 |  |  | 89 | 7,E-03 |
| Pef1 | Q8BFY6 |  |  | 84 | 1,E-02 |
| Rgmb | Q7TQ33 |  |  | 82 | 2,E-02 |
| Ncan | P55066 |  |  | 82 | 4,E-02 |
| Csf1 | P07141 |  |  | 79 | 4,E-02 |
| Smap;1110004F10Rik | Q9R0P4;D6RI64 |  |  | 75 | 2,E-02 |
| Sema6d | Q76KF0 |  |  | 68 | 2,E-03 |
|  | Q9D9H8 |  |  | 64 | 8,E-03 |
| Bcan | Q61361 |  |  | 53 | 4,E-02 |
| Vcam1 | P29533 |  |  | 53 | 3,E-02 |
| Sgce | O70258 |  |  | 49 | 5,E-04 |
| Tnc | Q80YX1 |  |  | 48 | 6,E-03 |
| Nrcam | Q810U4 |  |  | 37 | 6,E-05 |
| Xylt1 | F8VPK6;Q811B1 |  |  | 37 | 1,E-02 |
| Pxdn | Q3UQ28 |  |  | 35 | 4,E-02 |
| Mdga1 | D3Z499 |  |  | 34 | 4,E-02 |
| Stau2 | Q8CJ67 |  |  | 33 | 7,E-03 |
| Neo1 | P97798 |  |  | 31 | 1,E-02 |
| Cspg4 | Q8VHY0 |  |  | 31 | 6,E-03 |
| Pcdh1 | Q8CFX3;F7BJK1 |  |  | 30 | 3,E-02 |
| Zgrf1 | Q0VGT4;E9Q2K6 |  |  | 30 | 4,E-02 |
| Txndc16 | Q7TN22 |  |  | 28 | 4,E-02 |
| Robo1 | G5E843;O89026 |  |  | 23 | 2,E-02 |
| Prnp | P04925 |  |  | 23 | 8,E-04 |
| Mtif2 | Q91YJ5 |  |  | 21 | 2,E-02 |
| Aplp2 | Q06335 |  |  | 18 | 3,E-02 |
| Chl1 | P70232 |  |  | 17 | 3,E-02 |
| Cdv3 | Q4VAA2 |  |  | 15 | 1,E-03 |
| Lrfn4 | Q80XU8 |  |  | 15 | 5,E-05 |
| Ptprf | A2A8L5 |  |  | 13 | 2,E-02 |
| Sez6 | Q7TSK2 |  |  | 13 | 4,E-02 |
| Hmgn2;Gm16494 | A3KGL9; E9Q2Z4 |  |  | 12 | 1,E-02 |
| Podxl2 | F6TE23 |  |  | 12 | 1,E-02 |
| Hn1 | P97825 |  |  | 12 | 3,E-02 |
| Nfasc | Q810U3 |  |  | 12 | 4,E-02 |
| Ptprz1 | B9EKR1 |  |  | 10 | 4,E-03 |
| Lrrc4b | P0C192 |  |  | 10 | 2,E-02 |
| Tmem132b | F7BAB2 |  |  | 10 | 6,E-03 |
| Lamc1 | F8VQJ3;P02468 |  |  | 8 | 8,E-03 |
| Cdh2 | P15116;D3YYT0 |  |  | 8 | 1,E-02 |
| Bcas1 | Q80YN3 |  |  | 8 | 5,E-02 |
| Zfp428;Znf428 | H3BL05;H3BLD9 |  |  | 3 | 2,E-02 |

**Table S1: Proteins enriched in the medium in mock-infected control and HSV-1 infected primary cortical cultures.** Proteins are listed which were more abundant in the medium supernatant than in the cell-pellet (Fold Change greater than 2, p-value smaller than 0.05).
